# Chronic stage magnetic resonance imaging findings in patients with shoulder injury related to vaccine administration (SIRVA)

**DOI:** 10.1007/s00256-023-04334-3

**Published:** 2023-04-03

**Authors:** Ricardo Donners, Julian Gehweiler, Balazs Kovacs, Hanns-Christian Breit, Thomas Daikeler, Dorothee Harder, Christoph T. Berger

**Affiliations:** 1 Department of Radiology, University Hospital Basel, Petersgraben 4, CH-4031 Basel, Switzerland; 2 Rheumatology Clinic, University Hospital Basel, Basel, Switzerland; 3 University Centre for Immunology, University Hospital Basel, Basel, Switzerland

**Keywords:** Shoulder injury related to vaccine administration (SIRVA), MRI, Shoulder joint

## Abstract

**Purpose:**

Identify chronic shoulder MRI findings in patients with known shoulder injury related to vaccine administration (SIRVA).

**Materials and methods:**

Two fellowship-trained musculoskeletal radiologists retrospectively reviewed the MRI of nine patients with clinically established SIRVA. MRI was performed at least 4 weeks after vaccination and included intravenous contrast-enhanced sequences. MRI was reviewed for the presence of erosions, tendonitis, capsulitis, synovitis, bone marrow oedema, joint effusion, bursitis, cartilage defects, rotator cuff lesions, and lymphadenopathy. The number and location of focal lesions were recorded.

**Results:**

Erosions of the greater tuberosity were present in 8/9 (89%), tendonitis of the infraspinatus muscle tendon in 7/9 (78%), capsulitis, synovitis, and bone marrow oedema in 5/9 (56%) cases, respectively. Effusion was found in three, and subdeltoid bursitis, rotator cuff lesions as well as cartilage defects in one patient, respectively. None of our included subjects showed axillary lymphadenopathy.

**Conclusion:**

In this case series, greater humeral tuberosity erosions, infraspinatus muscle tendonitis, capsulitis, synovitis, and bone marrow oedema were common MRI findings in chronic SIRVA.

## Introduction

The COVID-19 pandemic has brought vaccinations back to the center of attention for global health. The safety of prophylactic vaccines is critical, especially in the context of worldwide mass immunization campaigns. Vaccinations are generally very safe interventions, and serious adverse events following immunization are rare [1]. Common local and minor systemic reactions, such as swelling, pain, myalgia, and fever, are a consequence of the vaccines’ mechanisms of action, i.e., immune activation, and are summarized as local or systemic “reactogenicity.”

In rare cases, local pain can be severe and persist over weeks with limited limb function. In this scenario, shoulder injury related to vaccine administration (SIRVA) has to be excluded. SIRVA summarizes injuries to the shoulder joint or surrounding tissues that are typically considered to be caused by vaccine misapplication [2]. While the pathogenesis is not yet fully understood, injection into the bursa or intrasynovial vaccine deposition appear to trigger a prolonged autoimmune response, targeting extracellular matrix proteins, which results in bursitis and chronic joint inflammation [2]. General awareness of SIRVA among physicians is low, making it an underdiagnosed condition [3]. Consequently, the prevalence and incidence of SIRVA are unknown, but it is likely a rare disease with an incidence well below 1% [4]. Nonetheless, with global immunization campaigns, it became evident that awareness of SIRVA and training of personnel to prevent SIRVA are critical [5].

SIRVA was reported with a variety of vaccines, most commonly after the frequently applied influenza and tetanus vaccines [6], but also following COVID-19 vaccinations [5, 7]. The diagnosis is usually made clinically, when shoulder pain persists for more than 3 days after vaccination in a previously unremarkable joint. Pain at rest and limited range of motion are typical for SIRVA. Nightly pain and shoulder weakness are also common findings. These symptoms occur in the acute stage, but often persist for more than 4 weeks and were reported even 32 weeks after vaccination [2]. Pathological findings in SIRVA include subacromial bursitis, tendonitis, myositis, bone erosions, and osteonecrosis. These can be diagnosed with high accuracy using magnetic resonance imaging (MRI) [2, 8, 9]. With the ever-improving global availability of MRI scanners as well as a general increase in medical imaging, radiologists should be familiar with typical SIRVA-related MRI findings. This allows them to potentially guide clinicians to the correct diagnosis in an appropriate clinical setting and avoid unnecessary extensive medical workup for differential diagnoses such as systemic inflammatory diseases.

Contemporary literature analyzing SIRVA MRI findings is limited to case reports and small case series comprising less than five patients with dedicated shoulder MRI in the acute phase [10]. MRI findings in SIRVA patients beyond the acute phase have not been described to date. Consequently, there is a need to review the MRI findings of chronic SIRVA, and the purpose of our study was to identify chronic shoulder MRI findings in patients with known SIRVA.

## Materials and methods

### Patients and inclusion criteria

This retrospective analysis of shoulder MRI findings was performed as part of an observational cohort of SIRVA cases, which was approved by the ethics and research committee of Northwestern and Central Switzerland (EKNZ 2017–01,726) and registered on ClinicalTrials.gov (NCT04236193). All patients provided written informed consent. The study population of this MR imaging case series includes the subjects from the previously published cohort that received an MRI [2] and four additional cases treated at our clinic, who initially presented with shoulder pain following vaccination, suggestive for SIRVA. The SIRVA diagnosis was established clinically in otherwise healthy patients with new shoulder pain following vaccination, persisting for more than 3 days in a previously asymptomatic joint.

Study inclusion criteria were, with the exception of SIRVA, otherwise healthy adult patients without pre-existing shoulder injury, a known time point of vaccine administration and the vaccine type, availability of diagnostic, intravenous (iv) contrast-enhanced shoulder MRI > 4 weeks and < 2 years after vaccine administration in our local picture archiving and communication system (PACS, Sectra, Sweden). Patients were excluded when clinical history and SIRVA diagnosis were equivocal, systemic, especially rheumatic disease was suspected or confirmed, or when iv contrast-enhanced MRI was not available.

### Imaging

MRI was performed on 3 T MRI (GE Discovery MR750, GE Healthcare, Chicago, USA, and Siemens Magnetom Skyra, Siemens Healthineers, Erlangen, Germany) systems. Gadolinium-based iv contrast (Dotarem ®, Guerbet, Villepinte, France) was applied in all patients (volume range 10–16 ml). Representative MRI-sequence parameters for the standard imaging protocol are shown in Table 1.

**Table 1:** MRI parameters

MRI sequence	T1	T2 FS	T2 FS	PD FS	T1 FS
Plane	Parasagittal	Parasagittal	Paracoronal	Axial	Axial
Slice thickness (mm)	3	3	3	2	3
Spacing (mm)	3.6	3.6	3.6	2.6	3.3
Flip angle	150°	150°	150°	150°	150°
Acquisition matrix (Px)	384*384	320*320	320*320	320*320	320*320
TR (ms)	762	4210	3400	5000	750
TE (ms)	11	72	75	34	11
Bandwidth (Hz/Px)	255	200	200	215	250
Acquisition time (min)	1:54	4:23	4:00	3:02	4:30

### MRI evaluation

Two board-certified, musculoskeletal (MSK) radiology fellowship-trained radiologists, with 10 and 8 years of MSK experience, respectively, re-read all MRI studies. Systematic re-reading was done in a single consensus reading session with immediate agreement on MRI findings.

Shoulder MR images were evaluated systematically regarding the presence, number, and location of a predefined set of potential findings, summarized in Table 2. “Presence” was used as a binary variable discriminating between the presence and absence of a finding.

**Table 2:** Image evaluation features

Feature	Analyzed parameters
Erosions	Presence, number (few–multiple–confluent), anatomic location
Bone marrow oedema	Presence
Humeral head necrosis	Presence
Cartilage defect	Presence, anatomic location, ICRS (International Cartilage Research Society) grade
Tendonitis	Presence, location (= affected muscle tendon)
Muscle oedema	Presence
Capsulitis	Presence
Synovitis	Presence, presence of pannus
Bursitis	Presence, location (= affected bursa)
Axillary lymphadenopathy	Presence
Rotator cuff lesion	Presence, location (= affected tendon)

Erosions were defined as sharply marginated bone defects, involving the cortical or subchondral bone and adjacent trabecular bone with loss of low signal intensity. Hyperenhancement on iv contrast-enhanced T1w FS images supported the diagnosis. When present, the anatomical location of erosions was noted. Moreover, the number of erosions was classified as few (1–2), multiple (> 2), or confluent (impossible to distinguish between single erosions).

Bone marrow oedema was defined as increased marrow signal on fluid-sensitive FS (T2w FS and PD FS) sequences with the effaced marrow-fat signal on the T1w sequence, in keeping with the glossary of terms for MSK radiology [11]. The term “oedema” is used equivalent to the term “oedema-equivalent signal” and does not imply a particular histopathology. Osteonecrosis was defined as a well-demarcated area with a reactive interface line and fatty center and preferably the presence of the double line sign on T2w FS images [11]. A cartilage defect was described when a focal lesion of the normally smooth chondral layer of the humeral head or glenoid was found. When present, cartilage defects were graded according to the International Cartilage Repair Society (ICRS) grading system [12]. The presence of tendonitis was defined as increased contrast enhancement and oedema-equivalent signal of and surrounding the affected muscle tendon. Likewise, capsulitis was defined as increased enhancement of the capsule and adjacent tissue on post-iv contrast T1w FS MRI with or without capsular thickening and surrounding oedema. Accordingly, synovitis was identified when the synovial layer showed increased enhancement on post-iv contrast T1-weighted FS MRI [11]. Focal synovial proliferations and the presence of pannus were noted separately. Bursitis was defined as increased fluid-equivalent signal in the affected bursa. Muscle oedema was defined as a diffuse increased signal of the affected muscle on fluid-sensitive FS images, when compared to the adjacent musculature. A rotator cuff tear was defined according to the whitepaper by Palmer et al. as a tendon disruption [11]. Axillary lymphadenopathy was described when an increased number of enlarged axillary lymph nodes was identified.

Reading, interpretation, and description of findings were performed including all available MRI sequences for each finding. Due to the study design, both readers were aware of the SIRVA diagnosis at the time of MRI interpretation.

### Statistics

Statistical analysis was performed using commercially available software (IBM SPSS Statistics Version 25, IBM Corp. Armonk, New York, USA) to derive median values and interquartile ranges.

## Results

### Cohort characteristics

We analyzed the MR images of nine SIRVA patients (7 women, 2 men), with a median age of 29.6 (IQR 28.75) years at the time of imaging. MRI was performed between November 2019 and April 2022. Diagnostic, contrast-enhanced shoulder MRI examinations were performed in all patients. The median time interval between vaccination and MRI was 35 (IQR 30) weeks. Seven patients received seasonal influenza vaccines, one patient tick-borne encephalitis (TBE) and one an mRNA COVID-19 vaccine, respectively.

### Imaging findings

MRI findings per patient, based on the consensus reading of the two MSK radiologists, are summarized in Table 3.

**Table 3:** MRI findings per patient

ID	Sex, age	Time to MRI (weeks)	Vaccine	Bone marrow oedema	Greater tuberosity erosion	Infraspinatus tendonitis	Effusion	Capsulitis	Synovitis	Bursitis
1	F, 50 yo	16	Influenca	Yes	Few	Yes	No	Yes	Yes	No
2	F, 56 yo	15	Influenca	Yes	Multiple	No	Yes	Yes	Yes	No
3	M, 25 yo	35	Influenca	Yes	Multiple	Yes	No	No	No	No
4	F, 23 yo	57	Influenca	No	Few	Yes	No	No	No	No
5	F, 26 yo	89	Influenca	No	Few	Yes	No	Yes	Yes	No
6	M, 49 yo	15	FSME	No	None	Yes	Yes	Yes	Yes	No
7	F, 67 yo	43	COVID Moderna	No	Few	No	Yes	Yes	Yes	Yes, subdeltoid
8	F, 22 yo	36	Influenca	No	Few	Yes	No	No	No	No
9	F, 30 yo	33	Influenca	No	Multiple	Yes	No	No	No	No

**Fig. 1:**
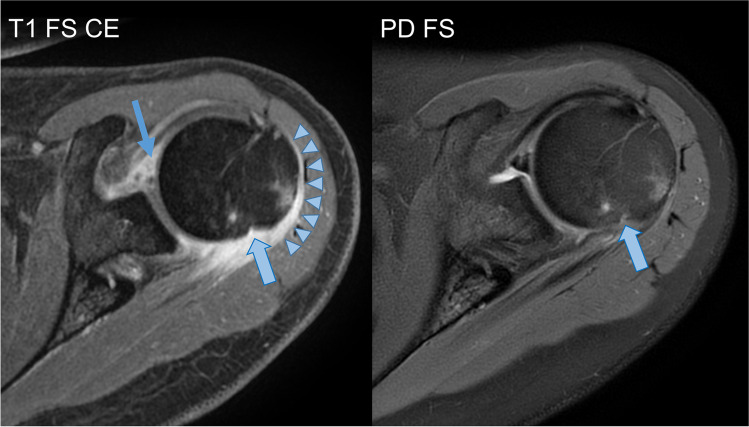
Capsulitis, synovitis and small erosion of the greater tuberosity in a 26-year-old female—capsulitis (arrow heads) and synovitis (solid arrow) identified as diffuse contrast enhancement, small erosion of the greater tuberosity (filled arrow), note how capsulitis and synovitis are better appreciated on T1-weighted fat-saturated (FS) contrast-enhanced (CE) MRI, with only little oedema on the PD-weighted FS image

**Fig. 2:**
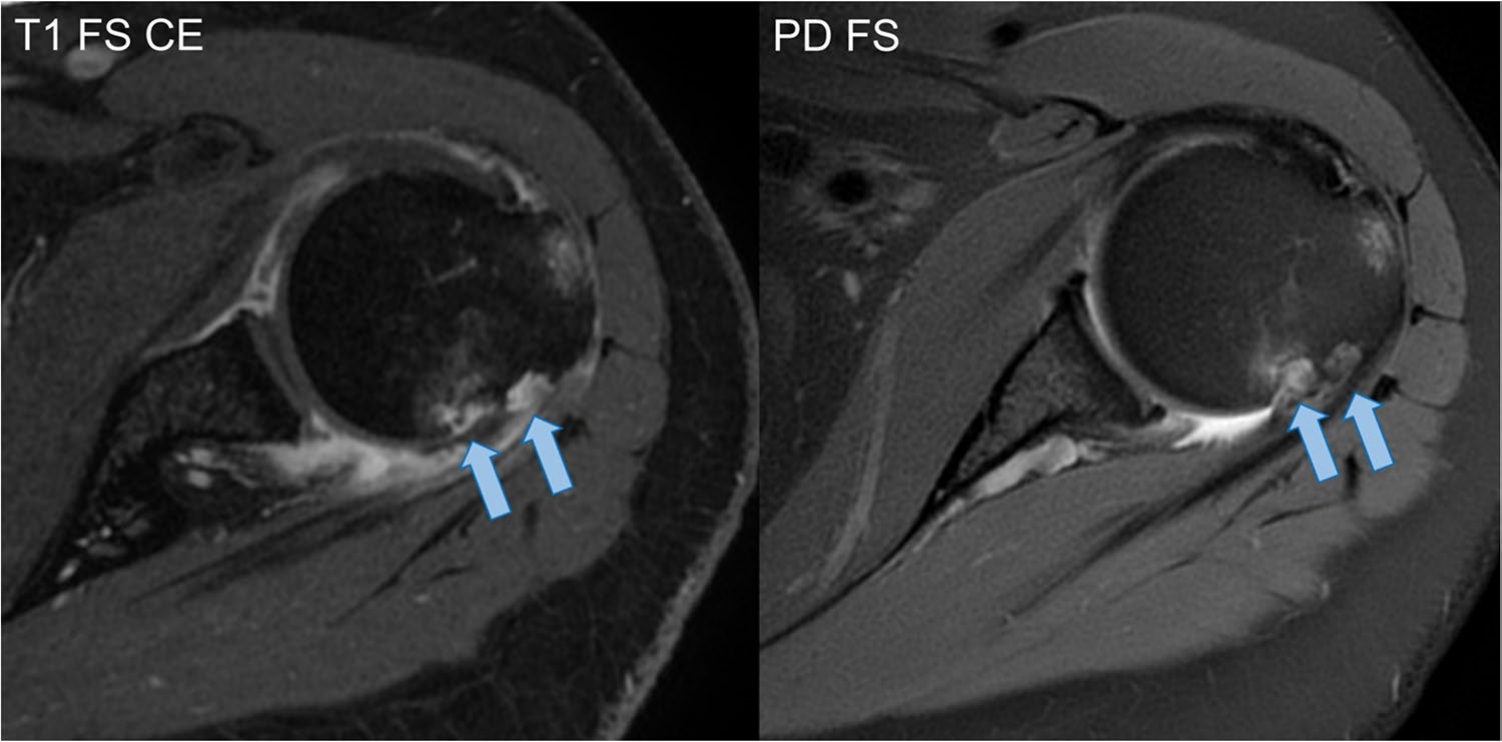
Multiple large erosions of the greater tuberosity of the humerus (arrows) in a 56-year-old female

Dedicated MRI review revealed erosions in 8/9 (89%) cases. Erosions, even when multiple, were exclusively located at the greater tuberosity of the humerus (Figs. 1 and 2).

**Fig. 3:**
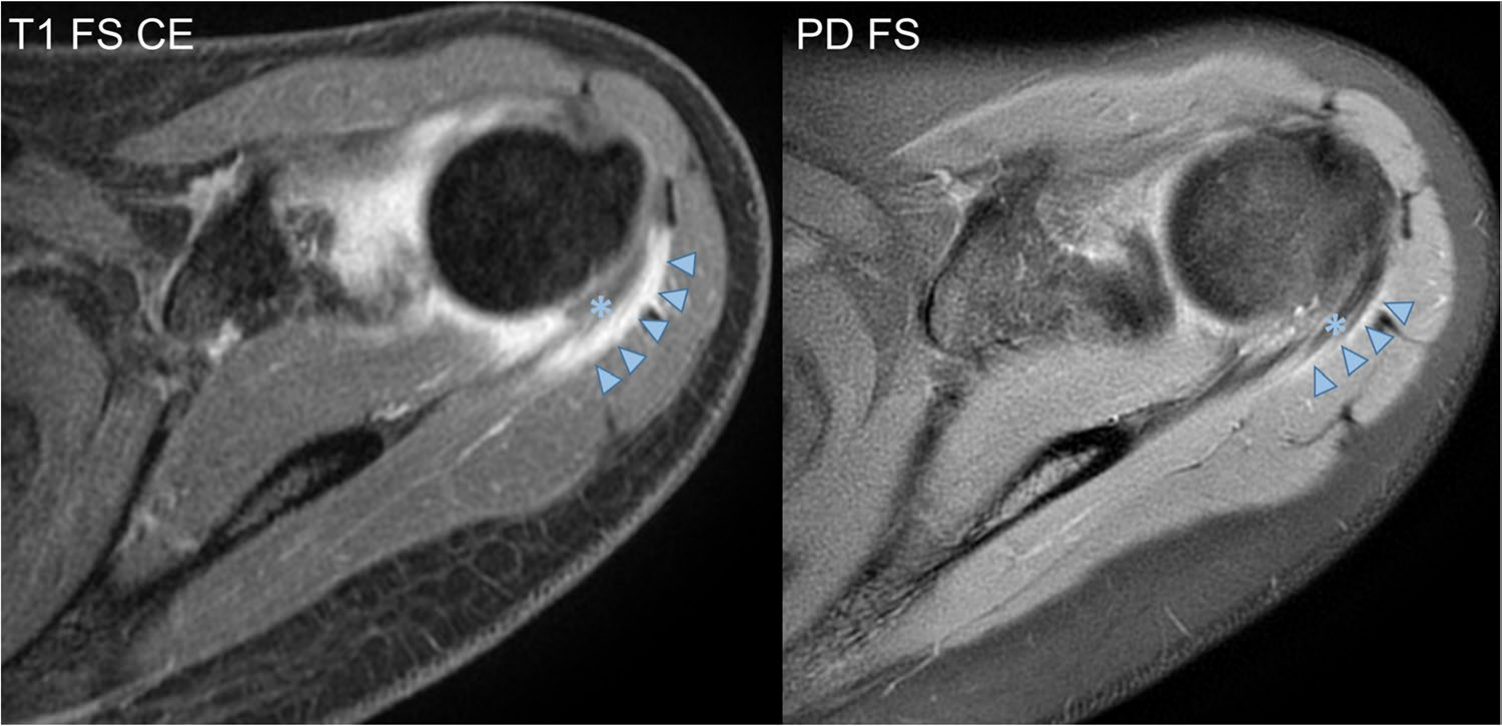
Infraspinatus tendonitis in a 22-year-old female—on the T1-weighted fat-saturated contrast-enhanced (CE) image, avid enhancement is evident (arrowheads) surrounding the M. infraspinatus tendon (*); by comparison, subtle oedema is present surrounding the tendon on the fluid-sensitive, fat-suppressed image (PD FS)

Tendonitis of the infraspinatus muscle tendon was identified in the majority of cases (7/9, 78%) (Fig. 3). The other tendons of the rotator cuff did not show inflammatory changes. Capsulitis and synovitis were reported in 5/9 cases (56%). Focal synovial proliferations or pannus were not identified. Bone marrow oedema of the humeral head was also found in 5/9 (56%) patients. No humeral head osteonecrosis was identified among the study subjects. A single patient showed a cartilage defect of the glenoid (ICRS grade III). Glenohumeral joint effusion was present in three cases (33%) and inflammatory changes of the subdeltoid bursa in a single case (11%). None of our included subjects showed evidence of axillary lymphadenopathy. A single patient showed a rotator cuff lesion, more specifically a partial tear of the supraspinatus tendon.

**Fig. 4:**
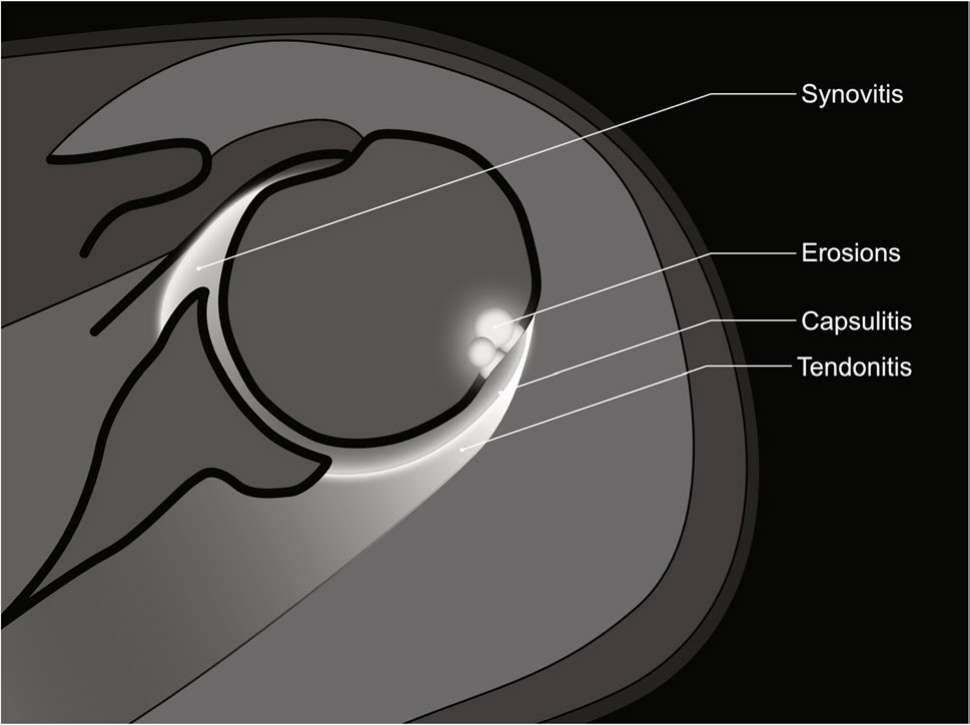
Typical, findings in chronic SIRVA on iv contrast-enhanced T1-weighted fat-saturated MRI

Based on the presented study cohort, common SIRVA iv contrast-enhanced MRI findings are shown in a pictogram (Fig. 4).

**Fig. 5:**
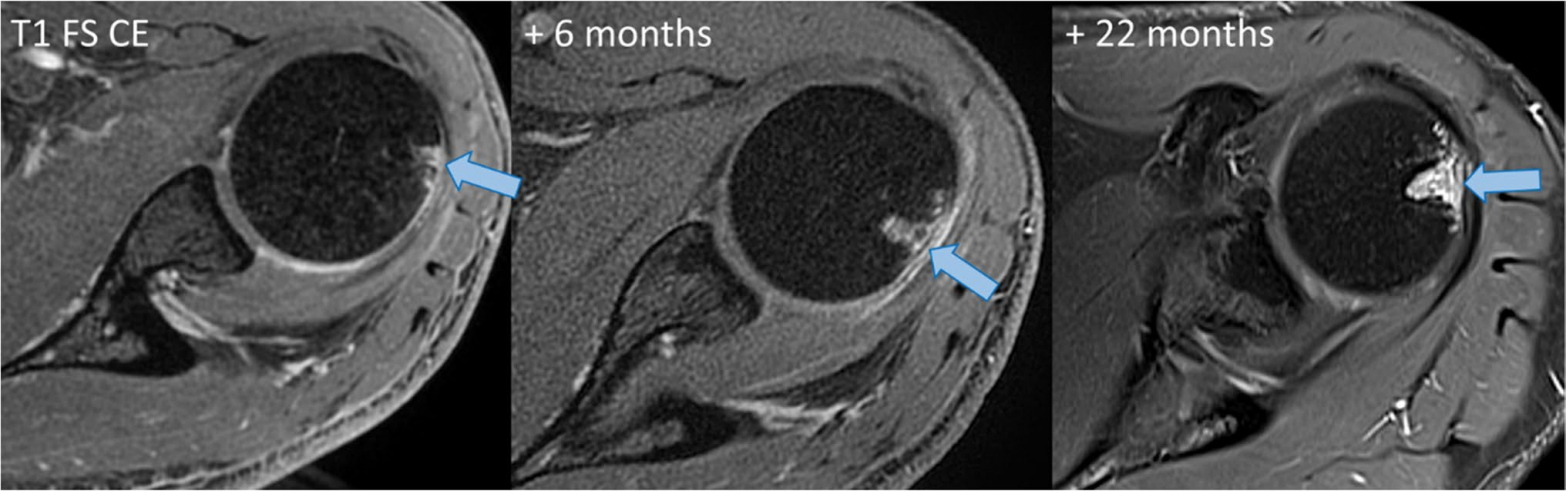
Evolution of greater tuberosity erosion on T1-weighted contrast-enhanced (CE) MRI from initial imaging to 6 months and an additional 22 months following initial MRI in a 25-year-old male, increase in the size of the greater tuberosity erosion (blue arrow) is shown

Follow-up MRI was available in two patients (patient ID 3 and 7). While one patient had a single follow-up MRI within 4 months that did not show significant change, patient #3 was followed up over 2 years with consecutive contrast-enhanced MRIs showing an increase in the size of the greater humeral tuberosity erosion (Fig. 5).

## Discussion

In this case series, we found that greater tuberosity erosions and tendonitis of the infraspinatus are very common MRI findings (89% and 78% prevalence, respectively) in chronic SIRVA. Capsulitis, synovitis, and bone marrow oedema were prevalent in more than 50% of cases, respectively.

SIRVA is defined as the onset of shoulder pain within 48 h of vaccination, typically also at rest, and most often limited range of motion. These symptoms persist for more than 3 days, commonly more than 4 weeks, in patients without any history of shoulder pain, inflammation, or joint dysfunction prior to vaccine administration [2]. While the pathogenesis is not fully elucidated, we recently reported an in-depth study on SIRVA immunopathogenesis. We found that the antigen deposition in the bursa or synovial space triggered a prolonged inflammatory reaction associated with the emergence of an autoimmune response targeting extracellular matrix proteins [2]. Potentially, the triggered autoimmune reaction may explain the presence of inflammatory findings for months after vaccination.

There is limited data available in the literature to compare our findings to previous analyses. To our knowledge, the largest case series describing MRI findings in SIRVA comprises four patients, which had all received the influenza vaccine [10]. MRIs were performed 7 days, 9 days, 20 days, and 3 months following vaccine administration, respectively. Among these patients, subdeltoid bursal fluid and bone marrow oedema were reported in 2/4 (50%) shoulder MRI, respectively [10]. Acquisition of iv contrast-enhanced images was not reported. Bursitis in the context of shoulder pain following vaccination has been recently discussed in a case report following COVID-19 vaccination [13]. In our original case series, we found bursitis in 4/16 subjects. Bursitis was mostly found, when ultrasound was performed early after vaccination [2]. Subdeltoid bursitis was only identified in a single patient in the presented MRI case series. Bursitis may be more indicative of the acute inflammatory phase of SIRVA. In the seminal paper that coined the term SIRVA, Atanasoff et al. described that unintentional vaccine administration in the subdeltoid bursa may cause bursitis, but also tendonitis [6]. This is supported by our findings, as tendonitis of the infraspinatus tendon was found in 78% of shoulder MRI. The involvement of the infraspinatus tendon is likely attributed to its anatomic location at the postero-inferior joint, close to the vaccine injection site.

In addition to the previously described SIRVA-associated findings such as bone marrow oedema, tendonitis, and capsulitis, our study gives a detailed description of erosions in the context of SIRVA. The majority of subjects (89%) showed erosions of the greater tuberosity of the humerus. This discrepancy to previous literature may be due to the longer vaccination-to-MRI time interval in our study. The median time between vaccine administration and MRI was 35 weeks in our study compared with 20 days in the previously cited 4-patient case series [10]. This may have given sufficient time for the development of erosions. Correspondingly, a case report found a greater tuberosity erosion at 3 months follow-up, which was not present on the initial MRI 2 days after the diphtheria–tetanus–poliomyelitis vaccination [14]. Salmon et al. hypothesized that the erosion may have been caused by direct vaccine-to-bone contact [14]. In a previous study, we found that the vaccine- and self-reactive T-cells as well as vaccine adjuvants can activate osteoclasts, resulting in bone resorption and possibly erosions in SIRVA. This was supported by the observation that SIRVA cases had increased serum levels of the osteoclast activation marker Tartrate-resistant acid phosphatase 5b (TRAP 5b) [2]. The local inflammatory reaction causing erosions may also be responsible for the bone marrow oedema of the humeral head.

In our study, we found that inflammatory changes of the shoulder joint, including capsulitis, synovitis, and tendonitis, are better appreciated on contrast-enhanced images when compared with fluid-sensitive FS MRI. Consequently, MRI in the context of SIRVA should be performed with iv contrast to aid detection of inflammatory findings and suggest the diagnosis in the appropriate clinical context. The importance of the knowledge of patient history needs to be emphasized. Although this study could identify findings that can suggest SIRVA, the most important information remains the clinical context: a previously healthy patient expressing shoulder pain for a prolonged time interval following vaccination. After all, erosions, tendonitis, capsulitis, synovitis, and bone marrow oedema are non-specific indicators of joint inflammation. Knowledge of the patient vaccination history can avoid extensive and expensive patient work-up regarding systemic-inflammatory differential diagnoses such as rheumatoid arthritis, axial spondylarthropathies, crystal deposition diseases or septic arthritis.

This case series has limitations, first and foremost the small patient number. Nonetheless, this study represents the largest MRI SIRVA cohort, and the presented common findings should guide radiologists to suggest SIRVA in the context of the appropriate patient history. Second, the MRI study was performed retrospectively, and we might have been biased due to our local high awareness for SIRVA, caused by the previously performed investigative study [2]. Third, subjects were vaccinated with different vaccines, but strongly biased towards the quadrivalent influenza vaccine. A review of available case reports has revealed no difference in clinical nor imaging presentation of SIRVA between vaccines [2]. Fourth, a baseline MRI study at symptom onset was not performed; thus, we cannot prove that MRI findings are unequivocally SIRVA manifestations. However, we only included otherwise healthy patients without a history of shoulder impairment before the vaccination. Although a small level of uncertainty remains, it is most likely that MRI findings are a consequence of vaccine application. Lastly, the MSK radiologists were not blinded to the SIRVA diagnosis, potentially creating bias.

Concluding, in this case series, greater humeral tuberosity erosions, infraspinatus muscle tendonitis, capsulitis, synovitis, and bone marrow oedema were common findings in chronic SIRVA. These findings should alert the radiologist to a potential SIRVA diagnosis in an appropriate clinical context.

## Data Availability

Raw data were generated at the University Hospital Basel. Derived data supporting the findings of this study are available from the corresponding author R.D. upon reasonable request.
